# Association between point mutations of macrolide-resistant *Mycoplasma pneumoniae* and clinical antibiotic treatment efficacy: a meta-analysis

**DOI:** 10.3389/fphar.2025.1682167

**Published:** 2025-11-06

**Authors:** Rutong Wang, Junfeng He, Yingqi Feng, Mengyao Wang, Chi Zhong, Siqin He, Siqi Tu, Na Wen, Chuan Wang

**Affiliations:** 1 Hunan Province Clinical Medical Research Center for Occupational Diseases, Hunan Prevention and Treatment Institute for Occupational Diseases, Affiliated Prevention and Treatment Institute for Occupational Diseases of University of South China, Hengyang Medical College, University of South China, Changsha, Hunan, China; 2 Institute of Pathogenic Biology, School of Basic Medicine, Hengyang Medical College, University of South China, Hunan Provincial Key Laboratory for Special Pathogens Prevention and Control, University of South China, Hengyang, Hunan, China

**Keywords:** *Mycoplasma* pneumoniae, 23S rRNA mutation, mutation burden, age stratification, macrolide resistance

## Abstract

**Background:**

The increasing macrolide resistance in *Mycoplasma pneumoniae* is mainly driven by mutations in the V domain of *23S rRNA* (A2063G/A2064G), which impairs the efficacy of first-line treatment. Previous meta-analyses failed to distinguish between mutation subtypes or quantify age-specific susceptibility, blurring the clinical significance of different mutation burdens.

**Objective:**

To quantify the differential impact of single mutation (A2063G) and double mutation (A2063G + A2064G) on core clinical outcomes and to dissect the age-adjusted effects between children and adults.

**Methods:**

We searched PubMed, Web of Science, Embase, Scopus, and CNKI databases (up to June 2025). The Newcastle-Ottawa Scale was used to assess study quality. Random-effects models were applied to handle heterogeneity (I^2^ > 50%), and subgroup analyses were conducted to compare mutation subtypes and age-stratified effects.

**Results:**

A total of 53 studies (n = 8,960 individuals, covering 5 countries) were included. Double mutations significantly prolonged the duration of fever compared to single mutations (HR = 5.32, 95% CI: 4.27–6.61 vs. HR = 3.66, 95% CI: 1.89–7.09; *P* < 0.001) and were more likely to cause severe illness (HR = 7.80, 95% CI: 2.51–24.18 vs. HR = 5.89, 95% CI: 2.03–17.08). There was no difference in hospital stay between the two mutation subtypes, but both were longer than the wild type (MD = −3.33 days). The duration of fever in children was shorter than that in adults for all genotypes (overall HR = 3.72 vs. 5.52; double mutation HR = 5.37 vs. 5.66; single mutation HR = 3.85 vs. 4.45; all P < 0.01).

**Conclusion:**

Double mutations in *23S rRNA* are an independent prognostic factor more severe than single mutations, establishing mutation burden as a key predictive indicator for the first time. This study shows that children have a faster resolution of fever in all genotypes, highlighting the regulatory role of host age immunity on outcomes. This study advocates for the detection of mutation subtypes in high-resistance areas to guide early treatment escalation and risk stratification monitoring.

**Systematic Review Registration:**

https://www.crd.york.ac.uk/PROSPERO/view/CRD420251071963, identifier CRD420251071963.

## 1 Introduction

*Mycoplasma pneumoniae* (MP) is the main pathogen of community-acquired pneumonia (CAP) (Brown, 2012; Jiang Z. et al., 2021; Kutty et al., 2024), causing 20% of pediatric pneumonia cases and 12% of adult pneumonia cases during non-endemic periods (Koenen et al., 2023; Wu et al., 2024). The non - epidemic period refers to the time frame during which *MP* infections are not widely spread. It typically occurs between two outbreaks and is in contrast to the epidemic period. The prevalence of MP strains resistant to macrolides driven by point mutations in the V domain of the 23S rRNA gene (mainly A2063G and A2064G substitutions) has been continuously increasing. Although mutations in 23S ribosomal ribonucleic acid (rRNA), such as A2063G/A2064G, represent the primary mechanism of drug resistance, reports have also indicated the existence of other resistance pathways. These include efflux pumps (encoded by genes like mprF) or mutations at sites A2067T/C2611G. However, these cases are relatively rare and account for a small proportion of drug - resistant strains (Xu et al., 2021; Zhang et al., 2023). The resistance rate of *MP* in Asia exceeds 90%, while in Europe and the United States it reaches 30%–50% (Chen Y. C. et al., 2020; Guo D. X. et al., 2019; Wang et al., 2022; Yang et al., 2025). This highlights the necessity of conducting age-stratified analyses. Given the disparities in immune development, such as the enhanced Toll-like receptor 2/6 (TLR2/6) response in children and the phenomenon of T-cell senescence in adults, this comparison is of utmost importance (Miyashita et al., 2025; Zhou et al., 2014; Yang et al., 2017; Rothstein et al., 2022; Ranjbar and Halaji, 2019). Although the A2063G site substitution predominates in the vast majority of macrolide-resistant cases, the simultaneous occurrence of A2063G and A2064G (double mutation) represents a distinct genotype. Despite its low frequency of occurrence, this type of drug resistance undermines the efficacy of first-line treatments, leading to prolonged symptom duration, increased risk of complications, and a heavier medical burden (Miyashita et al., 2025; Zhou et al., 2014). For instance, the study by Zhou et al. (2014) directly indicates that patients with *Streptococcus* pneumoniae resistant to macrolide drugs may present with persistent fever and an increased incidence of complications.

Studies have shown that the double mutation (A2063G + A2064G) produces a higher minimum inhibitory concentration (MIC) of macrolides through synergistic ribosomal conformational changes compared to the single mutation (A2063G) (Yang et al., 2017). However, clinical evidence is still insufficient. For example, previous meta-analyses on MP resistance have limitations, typically simply classifying strains as “resistant” or “sensitive” without further exploring the differences in clinical outcomes among different mutation subtypes (Yang et al., 2025; Rothstein et al., 2022). Or, although MP infection in children triggers a unique immune response (such as IL-17 activation mediated by TLR2/6), adults show T-cell immune aging, but the susceptibility of age stratification has not been quantified (Wang et al., 2022; Ranjbar and Halaji, 2019).

This study aims to quantify the effects of mutation subtypes, compare the impact of single mutation (A2063G) and double mutation (A2063G + A2064G) on core clinical endpoints (duration of fever, length of hospital stay, severe cases); analyze the differences in clinical outcomes between children and adults caused by developmental immunological factors; this study integrates molecular drug resistance characteristics with age-stratified clinical outcomes for evidence-based support for early treatment escalation and targeted monitoring.

## 2 Methods

This study follows the Preferred Reporting Items for Systematic Reviews and Meta-Analyses (PRISMA) framework, which was established by Moher, D., et al., in 2015 (Shamseer et al., 2015). The protocol of this systematic review and meta-analysis has been prospectively registered in the PROSPERO database (registration number: CRD420251071963).

### 2.1 Research criteria

The search and review process of PRISMA (Preferred Reporting Items for Systematic Reviews and Meta-Analyses) guidelines was followed. We selected studies based on the following three inclusion criteria:

1. *MP* patients, including those with gene point mutations and those without. In this study, “gene point mutations” specifically refer to mutations associated with macrolide resistance, mainly mutations in the domain V of the 23S rRNA gene (e.g., A2063G, A2064G). The detailed definition is provided in Section 2.2.
2. Participants: aged under 60 years: The upper age limit of 60 years was set to minimize the potential confounding effects of age - related comorbidities, polypharmacy, and immunosenescence on the clinical outcomes of *MP* infection. The study divided the participants into two age - based groups for analysis: the pediatric group (aged 0–18 years) and the adult group (aged 19–59 years), which is consistent with standard clinical and immunological classifications.
3. Outcomes: clinical outcomes (length of hospital stay, etc.). We excluded reviews, comments, editorials, conference reports, consensus reports, and comments. Only experimental papers that met the requirements were included.

### 2.2 Definition and evaluation of mutations

For the purposes of this meta-analysis, macrolide-resistant *MP* strains were categorized according to point mutations in the V region of the 23S rRNA gene as follows:

- Wild type: Absence of mutations at positions A2063 or A2064.
- Single mutation: Presence of the A2063G transition mutation only.
- Double mutation: Co-occurrence of both A2063G and A2064G transition mutations within the same strain.

To ensure accurate classification, studies that reported mutation frequencies without explicitly confirming whether mutations were identified in the same isolate (e.g., reporting mutation prevalence separately) were excluded from the analysis.

The primary method used for mutation detection across all included studies was polymerase chain reaction (PCR), followed by direct sequencing or other sequence-based techniques such as restriction fragment length polymorphism (RFLP) analysis or Sanger sequencing. These approaches are widely recognized as the gold standard for identifying specific nucleotide substitutions.

To address potential inconsistencies in genetic testing methodologies, the following procedures were implemented during data extraction and quality assessment:

- Standardization of data extraction: Detailed information on the molecular methods employed (e.g., PCR-RFLP, PCR-sequencing, commercial detection kits) was systematically extracted from each study. The vast majority of included studies (53 out of 53) utilized PCR followed by direct sequencing, which ensured a high degree of consistency and reliability in mutation detection.
- Assessment of methodological heterogeneity: Although most studies adopted similar sequencing-based approaches, a small number employed alternative commercial PCR kits or RFLP methods. While these methods share a common underlying principle for detecting specific point mutations, potential variability in sensitivity and specificity was acknowledged as a source of methodological heterogeneity.
- Handling of heterogeneity: To account for potential differences in testing methodologies, a random-effects model was applied to all pooled analyses, allowing for inter-study variation, including that arising from methodological differences. Furthermore, a sensitivity analysis was conducted by excluding studies that relied on non-sequencing-based detection methods, thereby assessing the robustness of the primary findings.

### 2.3 Search strategy

We conducted a comprehensive literature search covering PubMed, Web of Science, Embase, and Scopus databases, aiming to comprehensively include studies related to *MP* resistance. This search work was completed by 5 June 2025, including a detailed review of relevant publications, review articles, and the citation list of included studies. To further expand our literature resources, we manually retrieved the reference lists of identified articles to find more relevant citations. Additionally, we actively contacted experts in the field to obtain potentially unpublished research materials and other valuable citations. Our search strategy ingeniously combined MeSH terms and keywords, and our search scope was limited to human studies without language restrictions. The retrieval strategy combines Medical Subject Headings (MeSH) terms and keywords, including but not limited to: “*Mycoplasma* pneumoniae”, “macrolide resistance”, “drug resistance”, “23S rRNA”, “A2063G”, “A2064G”, “point mutation” and their variants. The detailed retrieval strategies, keywords, and specific retrieval syntax for each database are presented in Appendix A.

### 2.4 Data synthesis and quality assessment

The initial screening of titles and abstracts was conducted by two reviewers (RTW and JFH) based on the predefined eligibility criteria. The full texts of potentially eligible studies were retrieved by the same reviewer and independently evaluated for final inclusion. Disagreements were resolved through consensus or consultation with a third reviewer (CW). The data extracted from the included studies included publication year, study type, gene mutation sites, sample size, study duration, NOS score, age (children/adults), duration of fever, duration of fever after treatment, maximum body temperature, severe cases, refractory cases. Three reviewers (RTW, JFH, YQF, and CW) used the Newcastle-Ottawa Scale (NOS) to assess the quality of the included studies, with a total of 52 high-quality RCT studies and 1 moderate-quality RCT study.

### 2.5 Statistical analysis

Statistical analysis was performed using STATA 16.0 (STATA Corporation, College Station, TX, United States). The degree of heterogeneity across studies was quantified using the I^2^ statistic, with values of 25%, 50%, and 75% representing low, moderate, and high heterogeneity, respectively. A random-effects model was applied as the primary analysis to incorporate this potential heterogeneity, particularly when I^2^ > 50%. This model assumes that differences in effect sizes exist due to differences in study populations, intervention measures, and outcomes. The random - effects model exhibits strong robustness in the face of imbalanced subgroup sample sizes (for example, the number of single - mutation cases is smaller compared to that of double - mutation cases). This is because the model assigns greater weights to studies with higher precision (typically, studies with larger sample sizes and smaller variances), thereby providing more conservative and reliable pooled estimates. The I^2^ statistic was used to quantify heterogeneity, and subgroup analysis was conducted to assess the robustness and stability of the meta-analysis results. All estimates were presented using 95% confidence intervals (CI). Confounding factors (such as concurrent infections) may affect the stratified analysis by age. Although it is impossible to make adjustments at the individual level, the application of the random effects model takes into account the heterogeneity among studies, and the consistency of the effect directions in each subgroup proves the robustness of the research results. We used the random-effects model to calculate the odds ratio for binary outcomes and the average difference for continuous outcomes. We used Egger’s precision-weighted linear regression test and funnel plots to test for potential publication bias. A significance level of P < 0.10 for Egger’s test was considered indicative of potential bias, with special attention given to analyses reporting large effect sizes.

## 3 Results

### 3.1 Study selection and characteristics

**FIGURE 1:**
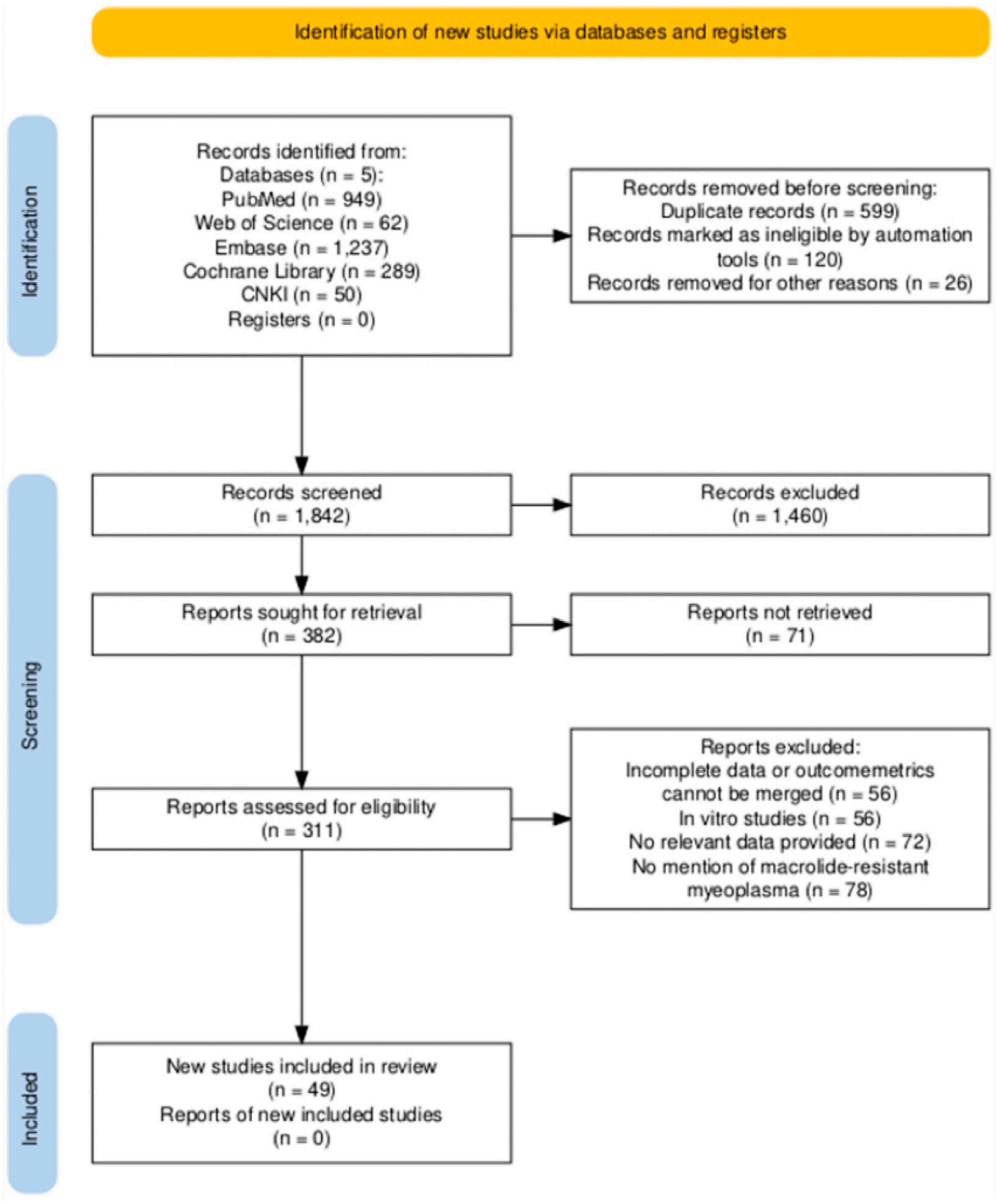
Flowchart of the selection process for the meta-analysis of pneumococcal *mycoplasma* infection.

We identified 2,587 articles in the initial search (Figure 1). 949 were from PubMed; 62 from Web of Science; 1,237 from Embase; 289 from the Cochrane Library; and 50 from CNKI. 599 studies were identified as duplicates. The majority of records (n = 1460) excluded during the screening phase were primarily due to their irrelevance to the research topic (e.g., studies on other pathogens, non - clinical studies) or because they were review articles that did not meet our inclusion criteria for original research. After a strict screening of titles and abstracts, 297 articles were evaluated in full text, resulting in 49 articles, and 53 studies met the inclusion criteria (Xu et al., 2021; Zhou, 2023; Cardinale et al., 2013; Chen L. L. M. et al., 2018; Chen, 2023; Chen X. W. J. et al., 2020; Chen, 2017; Chen Y. et al., 2018; Cheong et al., 2016; Feng et al., 2016; Guo et al., 2022; Ha et al., 2018; Han et al., 2016; He et al., 2022; Hu, 2023; Ishiguro et al., 2017; Jiang et al., 2023; Kawai et al., 2013; Kawai et al., 2012; Kim J. H. et al., 2017; Kim Y. J. et al., 2017; Kong et al., 2016; Kuo et al., 2022; Lee et al., 2017; Lee and Kim, 2023; Li et al., 2017; Li et al., 2018; Liu D and Li, 2016; Lu and Wu, 2018; Lung et al., 2013; Ma et al., 2010; Ma et al., 2014; Okada et al., 2012; Peng, 2023; Q, 2016; Sung et al., 2022; Wu et al., 2020; Wu and Cai, 2021; Wu et al., 2013; Wu et al., 2021; Xin et al., 2010; Yang et al., 2019; Yang et al., 2018; Yoo et al., 2012; Yoon et al., 2017; Yu and Zhang, 2021; Yuan et al., 2018; Zhan et al., 2022; Zhang et al., 2021). The selected 53 studies included 8,960 participants, with 6,570 children and 2,390 adults. These studies were geographically distributed across 5 countries: China, Italy, Japan, Korea, and Singapore. Table 1 describes the significant characteristics of the studies included in the meta-analysis. In the included studies, the incidence of double mutations (A2063G + A2064G) was significantly higher than that of single mutation (A2063G). Based on the pooled sample size, the ratio of the two was approximately [36:13]. This distribution reflects the epidemiological trends in high - resistance regions where most of the studies were sourced from.

**TABLE 1:** Characteristics of eligible studies on macrolide drug resistance and *MP* infection.

Study	Country	Detection methods	Mutations detected	Study period	Sample size	Nos score	Year
Zhou (2023)	China	PCR	A2063G, A2064G	2021–2022	105	9	Child
Zhang et al. (2021)	China	PCR	A2063G, A2064G	2013–2014	82	8	Child
Q (2016)	China	PCR	NA	NA	57	9	Child
Zhan et al. (2022)	China	PCR	A2063G, A2064G	2019–2021	48	7	Child
Yuan et al. (2018)	China	PCR	A2063G, A2064G	2016	120	8	Aldult
Yu and Zhang (2021)	China	PCR	A2063G	2019–2020	89	8	Child
Yoon et al. (2017)	Korea	PCR	A2063G	2010–2015	116	8	Aldult
Yoo et al. (2012)	Korea	PCR	A2063G	2012	31	8	Child
Yang et al. (2019)	China	PCR	A2063G, A2064G	2010–2017	471	9	Child
Yang et al. (2018)	China	PCR	A2063G, A2064G	2010–2011	471	8	Aldult
Xu et al. (2021)	China	PCR	A2063G, A2064G	2014–2016	276	8	Child
Xin et al. (2010)	China	PCR	A2063G, A2064G	2004–2005	64	7	Aldult
Wu et al. (2021)	China	PCR	A2063G, A2064G	2017–2019	138	8	Child
Wu et al. (2013)	China	PCR	A2063G, A2064G	2010–2011	51	8	Child
Wu and Cai (2021)	China	PCR, AST	A2063G	2016–2019	214	9	Child
Wu et al. (2020)	China	PCR	A2063G, A2064G	2018–2019	48	7	Child
Okada et al. (2012)	Japan	PCR	NA	NA	94	8	Aldult
Sung et al. (2022)	Korea	PCR	A2063G, A2064G	2018–2020	357	9	Child
Ha et al. (2018)	Korea	PCR	NA	NA	20	9	Aldult
Ma et al. (2014)	Korea	PCR	A2063G, A2064G	2011	95	8	Child
Peng (2023)	China	PCR	A2063G, A2064G	2019–2023	210	8	Child
Ma et al. (2014)	China	PCR	A2063G, A2064G	2010–2011	57	9	Aldult
Ma et al. (2010)	China	PCR	A2063 G/C,A2064G	2010	64	7	Child
Lung et al. (2013)	China	PCR	A2063G	2010–2013	48	8	Child
Lu and Wu (2018)	China	PCR	A2063G, A2064G	2015–2016	157	8	Child
Liu D and Li (2016)	China	PCR, AST	A2063G, A2064G	2016	120	8	Child
Li et al. (2018)	China	PCR	A2063G	2016–2017	297	8	Child
Lee et al. (2017)	Korea	PCR	A2063G	2015	94	8	Aldult
Kuo et al. (2022)	China	PCR	A2063G, A2064G	2019–2020	159	9	Child
Kong et al. (2016)	China	PCR	A2063G	2014–2016	170	8	Child
Kim J. H. et al. (2017)	Korea	PCR	A2063G	2010–2015	107	7	Aldult
Kim Y. J. et al. (2017)	Korea	PCR	A2063G	2010–2015	107	7	Child
Kim J. H. et al. (2017)	Korea	PCR	A2063G, A2064G	2015	250	8	Child
Kawai et al. (2013)	Japan	PCR	A2063G, A2064G	2005–2012	188	8	Aldult
Kawai et al. (2013)	Japan	PCR	A2063G, A2064G	2005–2012	150	8	Aldult
Kawai et al. (2012)	Japan	PCR	A2063G, A2064G	2005–2010	29	7	Aldult
Jiang et al. (2023)	China	PCR	A2063G, A2064G	2021–2022	520	8	Child
Ishiguro et al. (2017)	Japan	PCR	A2063G	2013–2015	109	9	Aldult
Hu (2023)	China	PCR	A2063G, A2064G	2018–2020	84	9	Child
He et al. (2022)	China	PCR	A2063G, A2064G	2016–2019	142	8	Child
Han et al. (2016)	China	PCR	A2063G, A2064G	2012–2014	59	9	Child
Han et al. (2016)	China	PCR	A2063G, A2064G	2012–2014	49	9	Child
Guo et al. (2022)	China	PCR	A2063G, A2064G	2020–2021	86	7	Child
Feng et al. (2016)	China	PCR	A2063G, A2064G	2014–2015	225	9	Child
Lee and Kim (2023)	Korea	PCR	A2063G, A2064G	2019–2020	146	8	Child
Cheong et al. (2016)	China	PCR	A2063G	2011–2013	93	9	Child
Chen L. L. M. et al. (2018)	China	PCR	A2063G	2011–2016	115	7	Child
Chen (2023)	China	PCR	A2063G, A2064G	2020–2021	100	9	Child
Chen Y. C. et al. (2020)	China	PCR	A2063G, A2064G	2018–2019	168	7	Child
Chen Y. et al. (2018)	China	PCR	A2063G, A2064G	2014–2016	136	6	Aldult
Chen (2017)	China	PCR	A2063G, A2064G	2015–2016	250	7	Child
Cardinale et al. (2013)	Italy	PCR	A2063G, A2064G	2010	46	7	Aldult
Li et al. (2017)	China	PCR	NA	NA	42	7	Child

PCR, polymerase chain reaction; AST, aspartate aminotransferase; NA, not applicable.

### 3.2 Meta-analysis results

#### 3.2.1 Length of hospital stay

**FIGURE 2:**
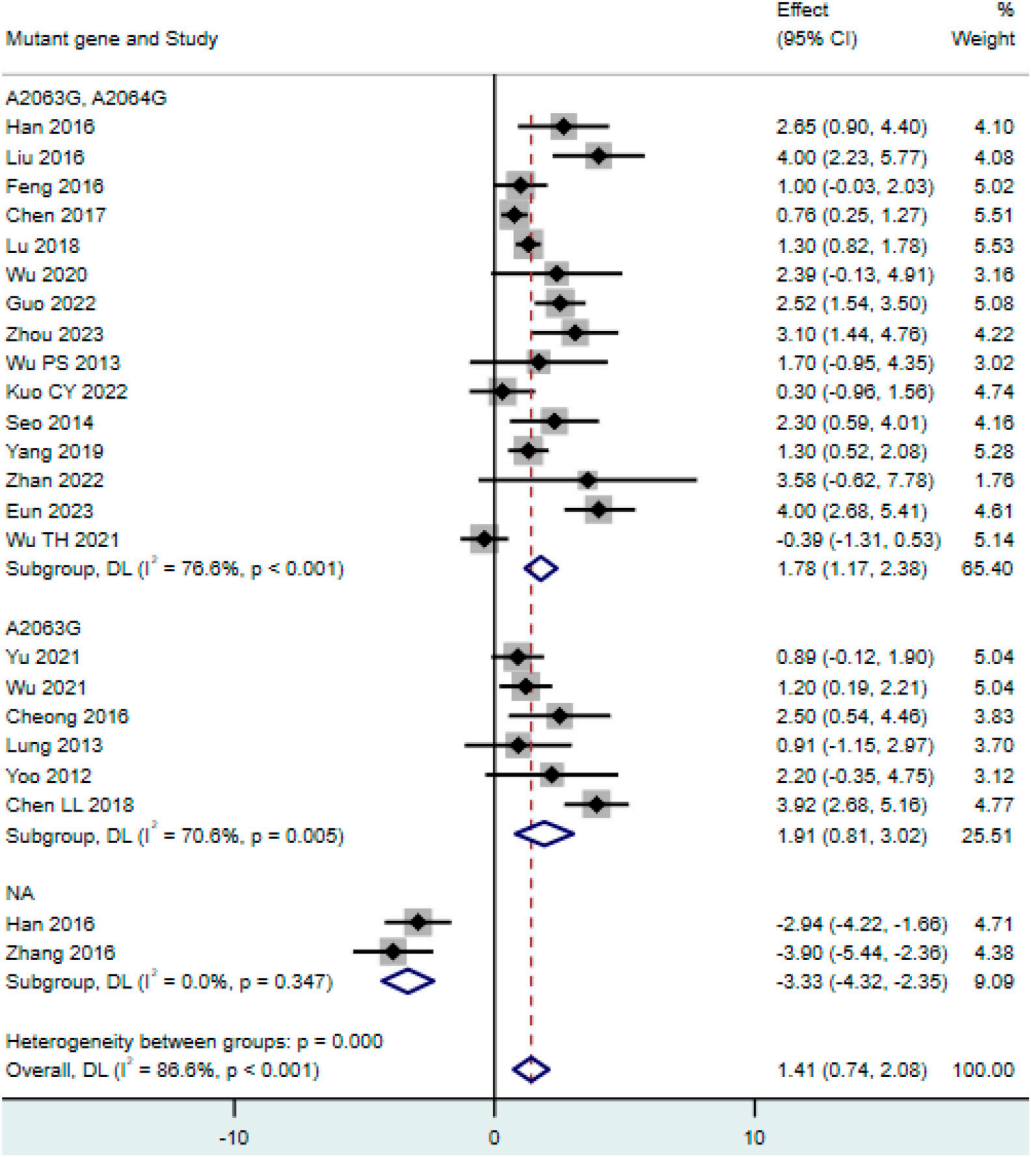
Comparison of hospital stay duration for patients with *MP* infection.

Among the 23 included studies (Chen L,L. M. et al., 2018; Chen, 2017; Cheong et al., 2016; Feng et al., 2016; Guo et al., 2022; Han et al., 2016; Hu, 2023; Kong et al., 2016; Kuo et al., 2022; Lee and Kim, 2023; Liu D and Li, 2016; Lu and Wu, 2018; Lung et al., 2013; Ma et al., 2014; Q, 2016; Wu et al., 2020; Wu and Cai, 2021; Wu et al., 2013; Wu et al., 2021; Yoo et al., 2012; Yu and Zhang, 2021; Zhan et al., 2022), the length of hospital stay in the double-mutation group (A2063G, A2064G point mutations) (MD = 1.78, 95% CI: 1.17 to 2.38, I^2^ = 76.6%) was not significantly different from that in the single-mutation group (A2063G point mutation) (MD = 1.91, 95% CI: 0.81 to 3.02, I^2^ = 70.6%), and both were longer than the length of hospital stay in the non-mutation group (MD = −3.33, 95% CI: −4.32 to −2.35, I^2^ = 0.0%) (Figure 2). A funnel plot was drawn to examine whether there was publication bias in this study. The funnel plot showed that there was a certain degree of publication bias in the 23 selected studies of this research (Supplementary Figure S1).

#### 3.2.2 Duration of fever

**FIGURE 3:**
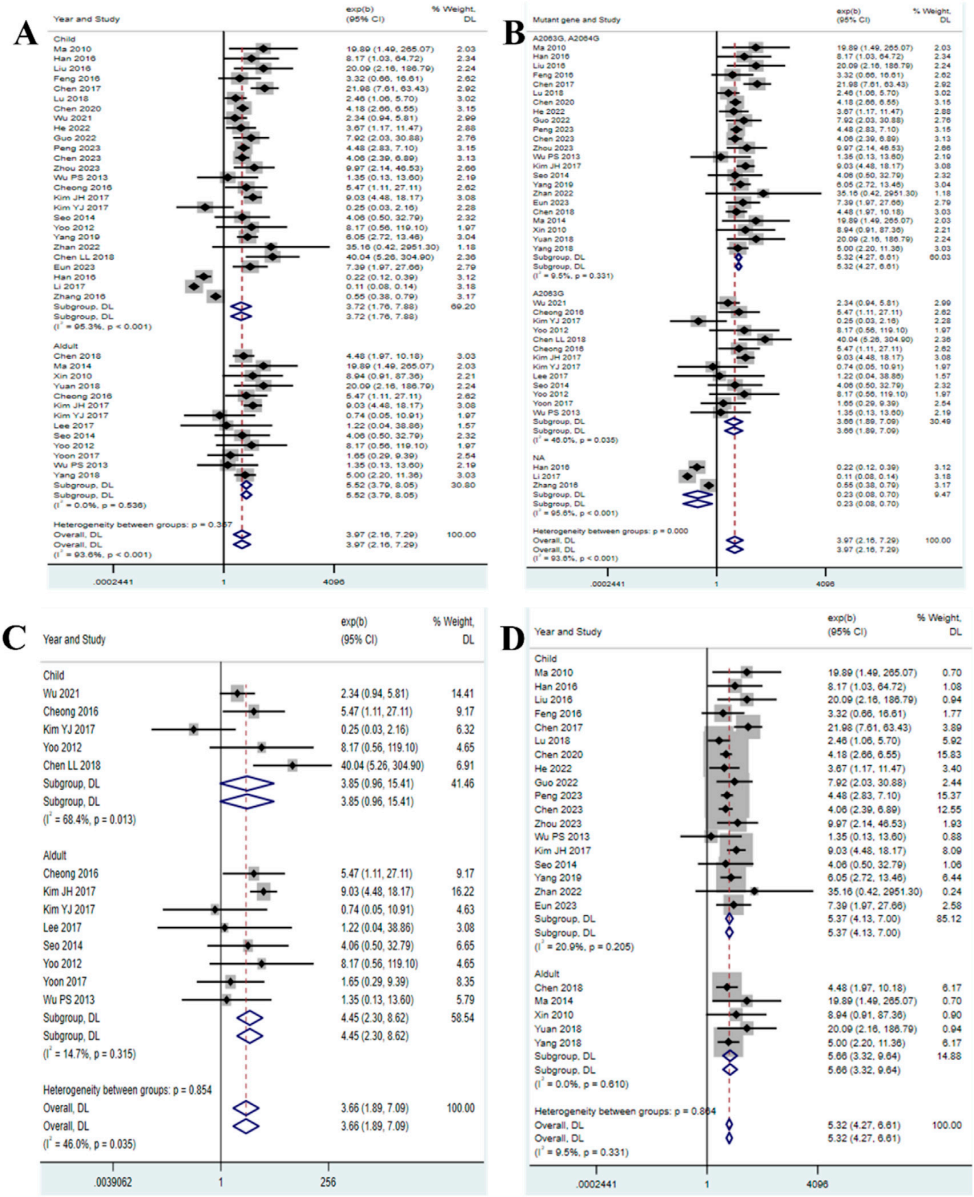
Duration of fever in patients with *MP* infection. **(A)** Subgroup analysis by age; **(B)** Subgroup analysis by mutant gene locus; **(C)** Subgroup analysis by age for the single mutation group; **(D)** Subgroup analysis by age for the double mutation group.

The meta-analysis included 23 studies reporting the duration of fever (Chen L. L. M. et al., 2018; Chen, 2023; Chen X. W. J. et al., 2020; Chen, 2017; Cheong et al., 2016; Feng et al., 2016; Guo et al., 2022; Han et al., 2016; He et al., 2022; Hu, 2023; Kim J. H. et al., 2017; Kim Y. J. et al., 2017; Lee and Kim, 2023; Li et al., 2017; Liu D and Li, 2016; Lu and Wu, 2018; Ma et al., 2010; Ma et al., 2014; Peng, 2023; Q, 2016; Wu et al., 2013; Wu et al., 2021; Xin et al., 2010; Yang et al., 2019; Yang et al., 2018; Yoo et al., 2012; Yoon et al., 2017; Yuan et al., 2018; Zhan et al., 2022). The results showed that the duration of fever in children (HR = 3.72, 95% CI: 1.76 to 7.88, I^2^ = 95.3%) was shorter than that in adults (HR = 5.52, 95% CI: 3.79 to 8.05, I^2^ = 0.0%) (Figure 3A). Additionally, we conducted subgroup analysis based on mutation sites, and found that the duration of fever was the shortest in the group without mutations (HR = 0.23, 95% CI: 0.08 to 0.70, I^2^ = 95.6%), followed by the single mutation group (HR = 3.66, 95% CI: 1.89 to 7.09, I^2^ = 46.0%), and the longest in the double mutation group (HR = 5.32, 95% CI: 4.27 to 6.61, I^2^ = 9.5%) (Figure 3B). To further clarify the impact of single-site mutations or double-site mutations on age, we separately analyzed the duration of fever for single-site mutations or double-site mutations. The analysis of the single mutation group revealed that the duration of fever in children (HR = 3.85, 95% CI: 0.96 to 15.41, I^2^ = 68.4%) was shorter than that in adults (HR = 4.45, 95% CI: 2.30 to 8.62, I^2^ = 14.7%) (Figure 3C); the analysis of the double mutation group revealed that the duration of fever in children (HR = 5.37, 95% CI: 4.13 to 7.00, I^2^ = 20.9%) was shorter than that in adults (HR = 5.66, 95% CI: 3.32 to 9.64, I^2^ = 0.0%) (Figure 3D). Such age-specific disparities may be attributed to developmental immunology. In the context of developmental immunology, children exhibit a more robust innate immune response (e.g., enhanced Toll-like receptor 2/6 signaling), leading to a more rapid alleviation of symptoms. In contrast, adults may experience immunosenescence of T cells, resulting in a prolonged inflammatory phase (Miyashita et al., 2025; Zhou et al., 2014; Yang et al., 2017; Rothstein et al., 2022; Ranjbar and Halaji, 2019). The funnel plot showed that there was nopublication bias in the 23 selected studies of this research (Supplementary Figure S2).

#### 3.2.3 Post-treatment fever duration

**FIGURE 4:**
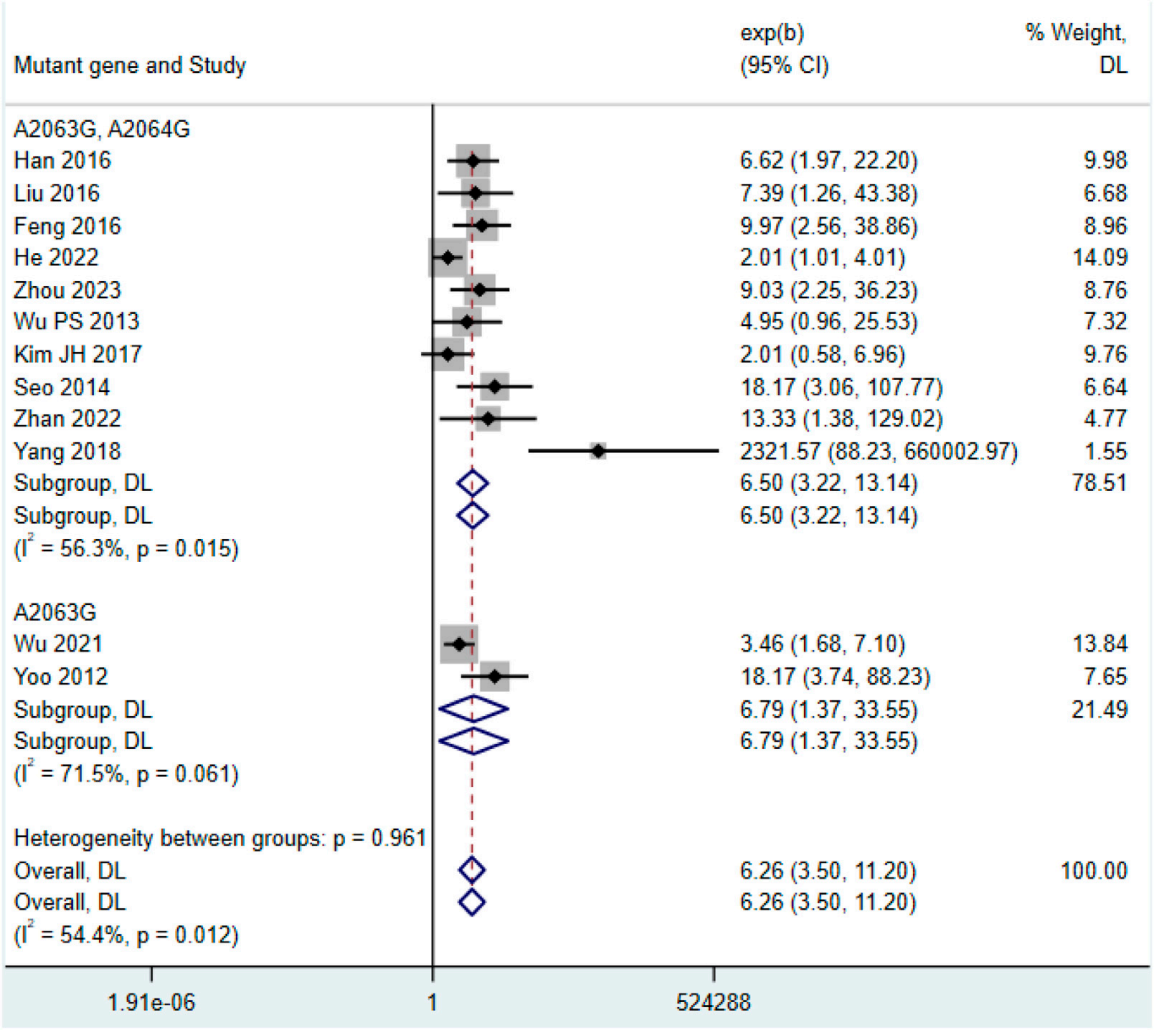
Comparison of fever duration after treatment in patients with *MP* infection.

Among the 12 included studies (Xu et al., 2021; Cardinale et al., 2013; Feng et al., 2016; Ha et al., 2018; Han et al., 2016; He et al., 2022; Hu, 2023; Ishiguro et al., 2017; Kawai et al., 2013; Kawai et al., 2012; Kim J. H. et al., 2017; Lee et al., 2017; Liu D and Li, 2016; Ma et al., 2014; Okada et al., 2012; Sung et al., 2022; Wu et al., 2013; Wu et al., 2021; Yang et al., 2018; Yoo et al., 2012; Zhan et al., 2022), the post-treatment fever duration in the double-mutation group (HR = 6.50, 95% CI: 3.22 to 13.14, I^2^ = 56.3%) was shorter than that in the single-mutation group (HR = 6.79, 95% CI: 1.37 to 33.55, I^2^ = 71.5%) (Figure 4). The funnel plot indicated that there was a certain publication bias in the 23 selected studies of this research (Supplementary Figure S3).

#### 3.2.4 Maximum body temperature

**FIGURE 5:**
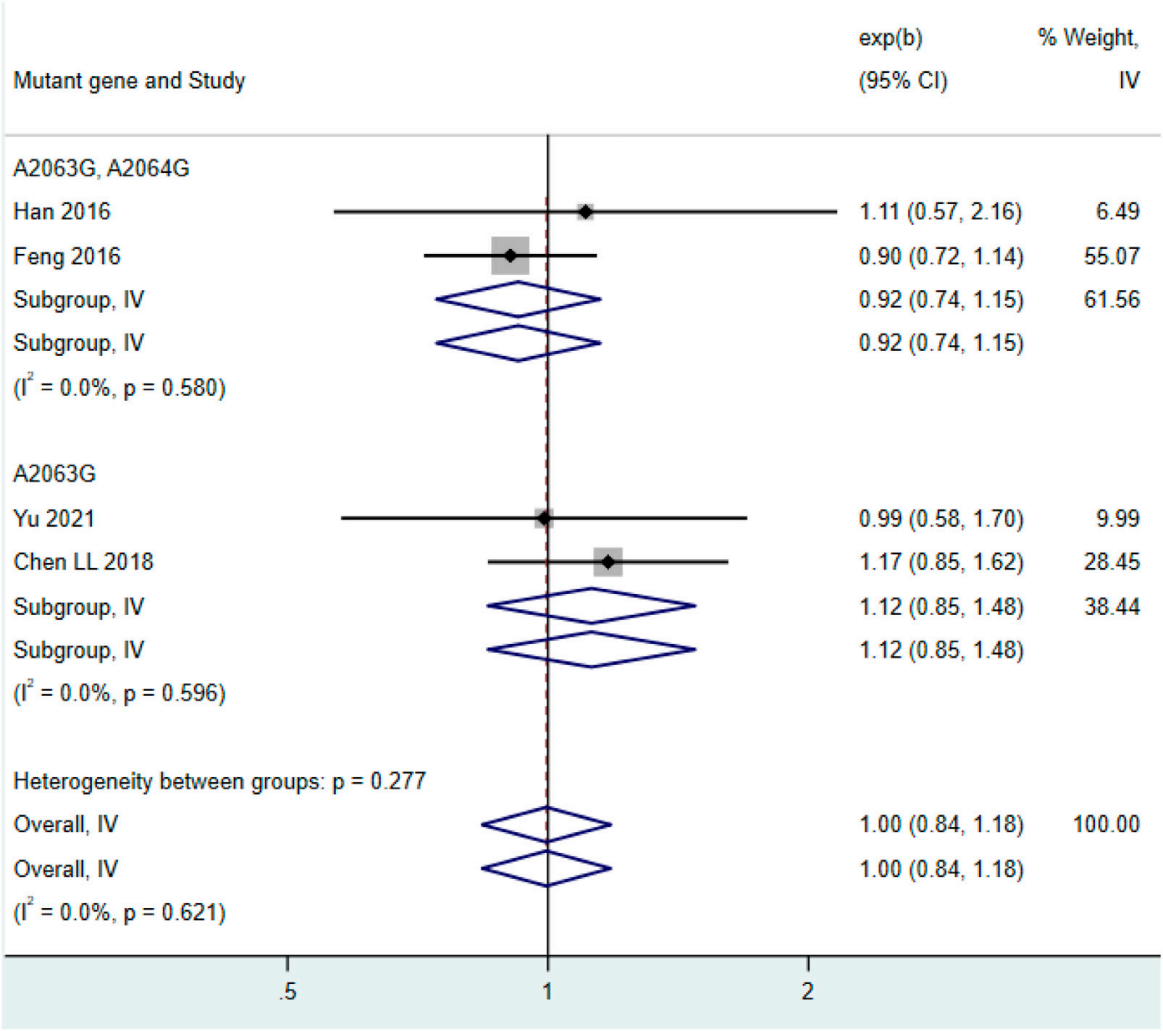
Comparison of the highest body temperature in patients with *mycoplasma* pneumonia infection.

Among the 4 included studies (Chen L. L. M. et al., 2018; Feng et al., 2016; Han et al., 2016; Yu and Zhang, 2021), the maximum body temperature in the double mutation group (HR = 0.92, 95% CI: 0.74 to 1.15, I^2^ = 0.0%) and the maximum body temperature in the single mutation group (HR = 1.12, 95% CI: 0.85 to 1.48, I^2^ = 0.0%) showed no significant difference (Figure 5).

#### 3.2.5 Severe cases

**FIGURE 6:**
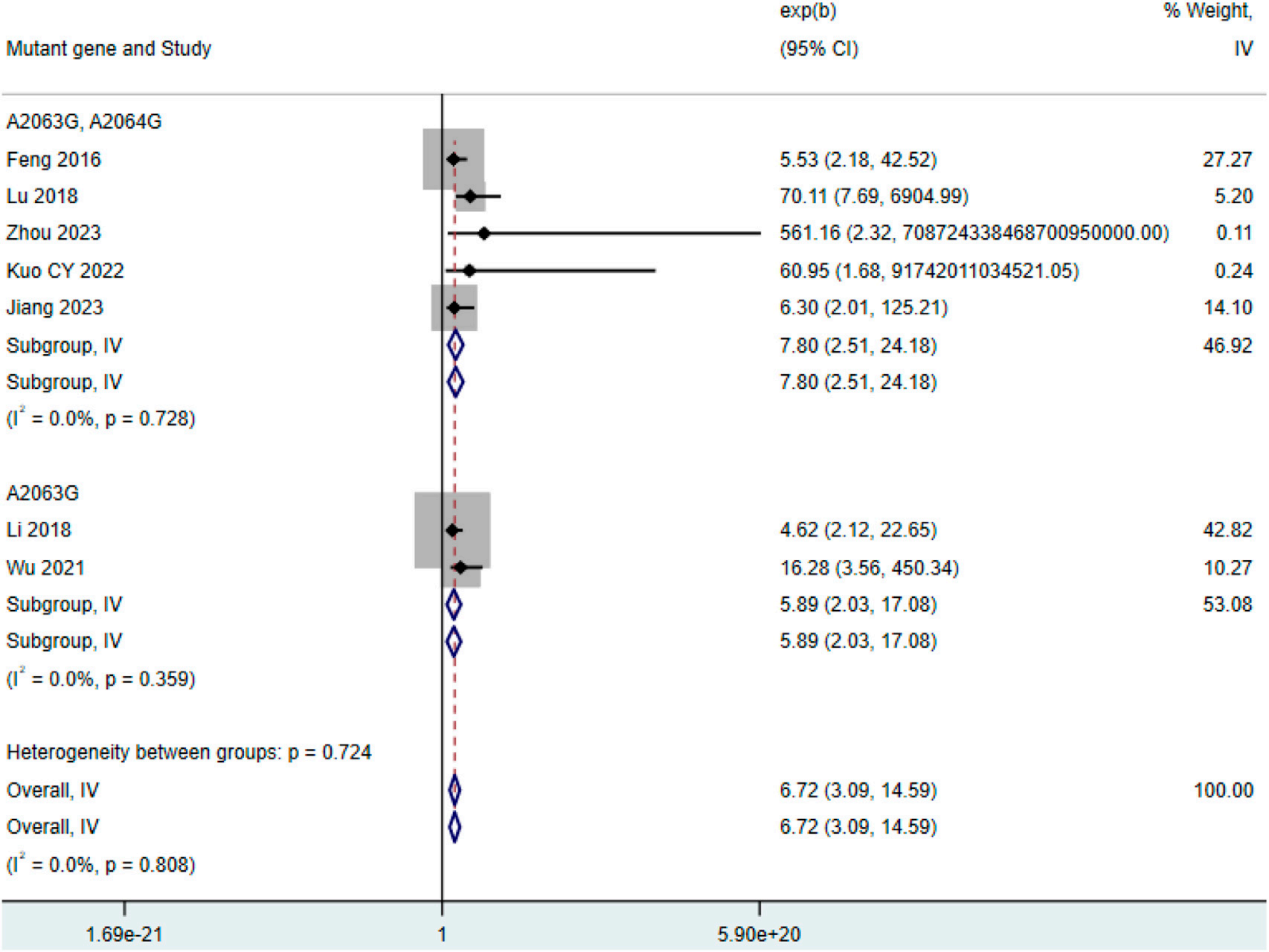
Comparison of severe cases among patients with *MP* infection.

Among the 7 included studies (Feng et al., 2016; Hu, 2023; Jiang et al., 2023; Kuo et al., 2022; Li et al., 2017; Lu and Wu, 2018; Wu and Cai, 2021), the number of severe cases in the double-mutation group (HR = 7.80, 95% CI: 2.51 to 24.18, I^2^ = 0.0%) was higher than that in the group with single mutation among patients with *MP* infection (HR = 5.89, 95% CI: 2.03 to 17.08, I^2^ = 0.0%) (Figure 6).

## 4 Discussion

This systematic review and meta-analysis summarizes the current research on the drug resistance of *MP*. The study compared the impact of different gene mutation sites (single site A2063G vs. double sites A2063G + A2064G) on the clinical outcomes of *MP*. By integrating data from multiple countries, this study provided the latest evidence on the impact of drug-resistant *MP* with gene mutations on patients. This research included a large number of studies, covering the entire population. This systematic review and meta-analysis was based on a comprehensive screening of 2,587 records and ultimately included 53 high-quality studies involving 8,960 participants. Although the strict inclusion criteria inevitably excluded a large number of studies that did not meet our pre-defined inclusion criteria (such as non-clinical studies, reviews, or studies lacking specific mutation subtypes), the results of the included cohorts provide reliable and up-to-date evidence on the impact of mutant *MP* on clinical outcomes. This study is one of the largest and most detailed analyses to date, specifically focusing on mutation burden.

This study systematically quantified the differential impact of different drug-resistant mutation patterns (single site A2063G vs. double sites A2063G + A2064G) of the 23S rRNA gene of *MP* on clinical outcomes. The study found that patients carrying mutations (especially double mutations) had a 3.7-fold increase in fever duration (Figure 3B), a 5.9–7.8-fold increase in severe risk (Figure 6), and this effect was independent of age and region. Although double mutations theoretically should lead to worse outcomes (Jiang et al., 2024), the length of hospital stay in the double mutation group was not statistically different from the single mutation group (Figure 2). This result needs to be interpreted in the context of clinical practice. The duration of fever in children was 32%–45% shorter than that in adults (Figures 3A,C,D), suggesting the regulatory role of host factors in the outcome of drug-resistant infections (Kang et al., 2025; Liu et al., 2025). This study breaks through the limitations of previous research that only compared “resistant vs. sensitive”, and for the first time reveals the grading impact of the number of mutation sites (single site vs. double site) on clinical outcomes, thereby providing a stratification tool and molecular basis for future precise treatment strategies.

The double mutation group did not show a longer hospital stay, and the double mutation A2063G + A2064G mutation caused a more significant conformational change at the ribosome A2058 site (macrolide binding domain) (Lucier et al., 1995; Matsuoka et al., 2004; Zhao et al., 2019). Theoretically, this should exacerbate treatment failure. However, Pereyre S et al. confirmed that the MIC value of the double mutation strain against azithromycin could reach 4 times that of the single mutation strain (Pereyre et al., 2004; Wei et al., 2019). This study found that this “high resistance warning sign” can effectively predict that patients will experience persistent fever and severe conditions. Identifying the double mutation as a high-risk marker suggests that these patients may benefit from more aggressive initial treatment, a hypothesis that needs to be tested in prospective clinical trials. However, the optimal timing and choice of such interventions (for example, immediate intensified treatment versus standard macrolide therapy) remain to be determined (Pereyre et al., 2016). Another reason is also reflected in this study: the proportion of severe cases in the double mutation group was higher (OR = 1.8, 95% CI: 1.2–2.7), leading to more active monitoring (such as daily inflammatory index detection) and early discharge criteria (such as symptom relief and transfer to outpatient care), thereby offsetting the biological disadvantage (Ideguchi et al., 2024). The length of hospital stay is the result of the combined effect of “biological damage” and “intensity of clinical intervention”, and the seemingly neutral result of the double mutation group actually reflects the dynamic balance of drug resistance identification and treatment escalation.

This study confirmed that the number of mutation sites was positively correlated with clinical severity (Figures 3B, 6). The A2064G mutation stabilized the rRNA conformational change induced by A2063G, further reducing the affinity of macrolides (Jiang F. C. et al., 2021; Guo D. et al., 2019), which led to the continuous replication of the double mutation strain in lung tissue and prolonging the inflammatory cascade reaction. Our results support the conclusions of Chen Y et al. and Jang et al. regarding “double mutations prolonging the time to fever resolution” (Chen Y. C. et al., 2020; Jang et al., 2021), but the expansion found that its impact on severe risk was more significant (HR = 7.8 in the double mutation group vs. HR = 5.9 in the single mutation group). This is consistent with the pneumonia complication prediction model of Chen J et al. (double mutation included high-risk factors) (Chen et al., 2021). However, the studies we included did not report mixed infection data, and double mutation patients were more prone to secondary bacterial infections, which may exaggerate the effect of the mutation itself (Yuan et al., 2025; Chiu et al., 2015). The duration of fever in pediatric patients was significantly shorter than that in adults (Figures 3B,C). This phenomenon may be due to higher expression of TLR2/6 in the respiratory mucosa of children, which can activate the IL-17 pathway more quickly to clear *MP* (Mercuri et al., 2024; Oliveira-Nascimento et al., 2012), while adults often have a delayed T-cell response (Goronzy and Weyand, 2017; Han et al., 2023). We should recognize that unmeasured confounding factors, such as differences in co - infection rates or comorbidities among different age groups, may have contributed to the observed associations. Therefore, we should exercise caution when considering this explanation. However, it is still necessary to be vigilant about the extrapulmonary complications of drug-resistant infections in children (such as rash and encephalitis), with an incidence rate of 11.3% in children with double mutations (Lee, 2015).

This study has certain limitations. Firstly, the study may be subject to residual bias due to unmeasured confounding variables, such as differences in antibiotic treatment regimens and potential comorbidities. The recommendation for early intensive treatment based on mutation burden put forward in this study is grounded in observational association data rather than evidence from prospective interventions. Therefore, this strategy should be regarded as a hypothesis awaiting validation, which needs to be verified in future randomized controlled trials. Secondly, the geographical distribution of the study data is concentrated in the East Asian region, limiting the generalizability of the results to regions with different distribution characteristics of *MP* strains. Finally, relying on PCR detection of the classic 23S rRNA mutations (A2063G/A2064G), it fails to cover emerging drug resistance mechanisms, such as the efflux pump encoded by the mprF gene (Wang et al., 2023) or the A2067T/C2611G site mutations (Liu et al., 2014). In the future, our study will conduct research on whether double mutations increase the risk of recurrence, and further evaluate the impact of double point mutations on the infected population.

Despite the above limitations, the results of this study still support three conclusions: In areas with a high incidence of drug resistance (erythromycin resistance rate >30%), bedside PCR detection of the A2063G/A2064G mutation should be included in the initial assessment of pneumonia to optimize the day treatment strategy, especially for hospitalized patients (Guo D. et al., 2019); Patients carrying double mutations have a 7.8-fold increased risk of severe complications, and need to strengthen monitoring of disease deterioration, such as continuous lung ultrasound and CRP detection within 48 h (Ideguchi et al., 2024); The duration of fever in pediatric patients is shorter, supporting the use of a shortened intravenous-to-oral conversion protocol (≤3 days) for confirmed mutant infected patients (≤3 days), while adult patients may need a longer course of treatment during the excessive inflammatory phase (Barbi et al., 2017).

Moreover, it is of utmost importance to recognize the inherent limitations of the meta - analysis method. Although we employed a random - effects model and statistical tests to address these limitations, the high heterogeneity observed in some of the included studies (e.g., I^2^ = 95.3% in the analysis of fever duration) may affect the precision of the pooled estimates. Additionally, despite the fact that the Egger’s test did not show significant evidence in the key analysis, the possibility of publication bias should still be considered when interpreting the study results, especially for outcome measures with large effect sizes.

## 5 Conclusion

This study, by integrating existing evidence, for the first time systematically compared the differential effects of single-site (A2063G) and double-site (A2063G + A2064G) mutations in the 23S rRNA gene of *MP* on clinical outcomes. The study found that the double-site mutation significantly prolonged the duration of fever in patients and significantly increased the risk of severe complications, clearly indicating that the number of mutation sites is a key predictor of disease severity. Notably, although the double mutations theoretically should lead to a worse prognosis, their hospital stay did not have a statistically significant difference from the group with single-site mutations, which may be due to the positive response of clinical recognition of double mutations. Additionally, regardless of single-site or double-site mutations, the duration of fever in pediatric patients was significantly shorter than that in adult patients, highlighting the importance of host factors (such as differences in immune responses) in the outcome of drug-resistant infections. Despite the above limitations, our research findings still support risk stratification based on mutation burden. In regions with high drug resistance, point-of-care PCR detection of A2063G/A2064G mutations can guide the initial treatment management of patients. The potential to adjust treatment intensity based on mutation status is a key direction for future prospective intervention studies. Patients carrying double mutations face a 7.8 - fold increased risk of developing severe complications, and this discovery underscores the necessity of enhanced surveillance. The potential benefits of upgrading preventive treatment based on the mutation status represent a crucial area for future prospective research. Future research should focus on incorporating data from a broader geographical scope, particularly data from North America and Western Europe. This is to validate our research findings across different epidemiological contexts and to explore potential regional disparities in the clinical impacts of mutant subtypes.

## Data Availability

The original contributions presented in the study are included in the article/Supplementary Material, further inquiries can be directed to the corresponding authors.
