# Supplementary material for: Association between point mutations of macrolide-resistant Mycoplasma pneumoniae and clinical antibiotic treatment efficacy: a meta-analysis
Source: Front Pharmacol. 2025 Nov 6;16:1682167. doi: 10.3389/fphar.2025.1682167 (PMC12631039; doi:10.3389/fphar.2025.1682167)
Supplement: Supplementary file 1 [file DataSheet1.zip › Supplementary files/Appendix A.docx]

**Appendix 1. Pubmed search strategy**

***Searched June 4, 2025***

#1 (((((Mycoplasma pneumoniae[MeSH Terms]) OR (Mycoplasma pneumoniae[Title/Abstract])) OR (M. pneumoniae[MeSH Terms])) OR (M. pneumoniae[Title/Abstract])) OR (pneumoniae, Mycoplasma[MeSH Terms])) OR (pneumoniae, Mycoplasma[Title/Abstract]) *(****9,977****)*

#2 ((((((((((((((((((((((((((((Drug Resistance, Microbial[MeSH Terms]) OR (Drug Resistance, Microbial[Title/Abstract])) OR (Drug Resistances, Microbial[Title/Abstract])) OR (Antibiotic Resistance, Microbial[MeSH Terms])) OR (Antibiotic Resistance, Microbial[Title/Abstract])) OR (Antibiotic Resistance[Title/Abstract])) OR (Antibiotic Resistance[MeSH Terms])) OR (Antimicrobial Resistances, Drug[MeSH Terms])) OR (Antimicrobial Resistances, Drug[Title/Abstract])) OR (23S rRNA[Title/Abstract])) OR (23S rRNA[MeSH Terms])) OR (RNA, Ribosomal, 23S[MeSH Terms])) OR (RNA, Ribosomal, 23S[Title/Abstract])) OR (23S Ribosomal RNA[Title/Abstract])) OR (23S Ribosomal RNA[MeSH Terms])) OR (Ribosomal RNA[MeSH Terms])) OR (Ribosomal RNA[Title/Abstract])) OR (rRNA gene[Title/Abstract])) OR (rRNA gene[MeSH Terms])) OR (A2063G[MeSH Terms])) OR (A2063G[Title/Abstract])) OR (A2064G[Title/Abstract])) OR (A2064G[MeSH Terms])) OR (C2617G[MeSH Terms])) OR (C2617G[Title/Abstract])) OR (ermB[Title/Abstract])) OR (ermB[MeSH Terms])) OR (mutation[MeSH Terms])) OR (mutation[Title/Abstract]) *(****1,430,508****)*

#3 #1 AND #2 *(****949****)*

**Appendix 2. Web of Science search strategy**

***Searched June 4, 2025***

#1 ((TS=(Mycoplasma pneumoniae)) OR TS=(M. pneumoniae)) OR TS=(pneumoniae, Mycoplasma) *(****24,2714****)*

#2 (((((((((((((((TS=(Drug Resistance, Microbial)) OR TS=(Drug Resistances, Microbial)) OR TS=(Antibiotic Resistance, Microbial)) OR TS=(Antibiotic Resistance)) OR TS=(Antimicrobial Resistances, Drug)) OR TS=(23S rRNA)) OR TS=(RNA, Ribosomal, 23S)) OR TS=(23S Ribosomal RNA)) OR TS=(Ribosomal RNA)) OR TS=(rRNA gene)) OR TS=(A2063G)) OR TS=(A2064G)) OR TS=(C2617G)) OR TS=(ermB)) OR TS=(mutation) *(****2,487,274****)*

#3 (TS=(RCT)) OR TS=(Randomized controlled trial) *(****830,303****)*

#4 #1 AND #2 AND #3 *(****62****)*

**Appendix 3. Embase search strategy**

***Searched June 4, 2025***

#1 'mycoplasma pneumoniae'/exp OR 'mycoplasma pneumoniae':ab,ti OR 'm. pneumoniae' OR 'm. pneumoniae':ab,ti OR 'pneumoniae, mycoplasma'/exp OR 'pneumoniae, mycoplasma':ab,ti *(****13,151****)*

#2 'drug resistance, microbial'/exp OR 'drug resistance, microbial':ab,ti OR 'drug resistances, microbial' OR 'drug resistances, microbial':ab,ti OR 'antibiotic resistance'/exp OR 'antibiotic resistance':ab,ti OR 'antimicrobial resistances, drug' OR 'antimicrobial resistances, drug':ab,ti OR '23s rrna':ab,ti OR 'rna, ribosomal, 23s':ab,ti OR '23s ribosomal rna':ab,ti OR 'ribosomal rna':ab,ti OR 'rrna gene':ab,ti OR a2063g:ab,ti OR a2064g:ab,ti OR c2617g:ab,ti OR ermb:ab,ti OR mutation:ab,ti *(****1,000,648****)*

#3 #1 AND #2 *(****1,237****)*

**Appendix 4. Cochrane search strategy**

***Searched June 4, 2025***

#1 ("Mycoplasma pneumoniae"):ti,ab,kw OR (M. pneumoniae):ti,ab,kw OR (pneumoniae, Mycoplasma):ti,ab,kw (Word variations have been searched) *(****3,323****)*

#2 (Drug Resistance, Microbial):ti,ab,kw OR (Drug Resistances, Microbial):ti,ab,kw OR (Antibiotic Resistance, Microbial):ti,ab,kw OR (Antibiotic Resistance):ti,ab,kw OR (Antimicrobial Resistances, Drug):ti,ab,kw (Word variations have been searched) *(****8,862****)*

#3 (RNA, Ribosomal, 23S):ti,ab,kw OR (23S rRNA):ti,ab,kw OR (23S Ribosomal RNA):ti,ab,kw OR (Ribosomal RNA):ti,ab,kw OR (rRNA gene):ti,ab,kw (Word variations have been searched) *(****1,795****)*

#4 (A2064G):ti,ab,kw OR (A2063G):ti,ab,kw OR (C2617G):ti,ab,kw OR (ermB):ti,ab,kw OR (mutation):ti,ab,kw (Word variations have been searched) (19851)

#5 #2 OR #3 OR #4 *(****30,003****)*

#6 #1 AND #5 *(****289****)*

**Appendix 5. CNKI**

***Searched June 4, 2025***

#1 肺炎支原体[主题词] AND 耐药性[主题词] AND 基因突变[主题词] *(****50****)*
