# Supplementary material for: Association between point mutations of macrolide-resistant Mycoplasma pneumoniae and clinical antibiotic treatment efficacy: a meta-analysis
Source: Front Pharmacol. 2025 Nov 6;16:1682167. doi: 10.3389/fphar.2025.1682167 (PMC12631039; doi:10.3389/fphar.2025.1682167)
Supplement: Supplementary file 1 [file DataSheet1.zip › Supplementary files/Supplementary Table 1 Quality assessment of the included studies.docx]

Supplementary Table 1 Quality assessment of the included studies

Selection Comparability Outcome

| Study | Represent  Ativeness of the exposed cohort | Selection of the nonexposed cohort | Ascertain  ment of exposure | Demonstration that outcome of interest was not present at start of study | Comparability of cohorts on the basis of the design or analysis | Assessment of outcome | Was follow-up long enough for outcomes to occur | Adequacy of follow up of cohorts | Quality score |
| --- | --- | --- | --- | --- | --- | --- | --- | --- | --- |
| Zhou 2023 | * | * | * | * | ** | * | * | * | *9* |
| Zhang 2021 | * |  | * | * | ** | * | * | * | *8* |
| Zhang 2016 | * | * | * | * | ** | * | * | * | *9* |
| Zhan 2022 | * |  | * |  | ** | * | * | * | *7* |
| Yuan 2018 | * |  | * | * | ** | * | * | * | *8* |
| Yu 2021 |  | * | * | * | ** | * | * | * | *8* |
| Yoon 2017 | * | * | * |  | ** | * | * | * | *8* |
| Yoo 2012 | * | * | * | * | ** | * | * |  | *8* |
| Yang 2019 | * | * | * | * | ** | * | * | * | *9* |
| Yang 2018 | * | * | * | * | ** | * | * |  | *8* |
| Xu 2021 | * |  | * | * | ** | * | * | * | *8* |
| Xin 2010 | * |  | * |  | ** | * | * | * | *7* |
| Wu TH 2021 | * |  | * | * | ** | * | * | * | *8* |
| Wu PS 2013 | * | * | * | * | ** | * | * | * | *8* |
| Wu 2021 | * | * | * | * | ** | * | * | * | *9* |
| Wu 2020 | * | * | * | * | ** |  | * |  | *7* |
| Takafumi Okada 2012 | * | * | * | * | ** | * |  | * | *8* |
| Sung 2022 | * | * | * | * | ** | * | * | * | *9* |
| Seok Gyun Ha 2018 | * | * | * | * | ** | * | * | * | *9* |
| Seo 2014 | * | * | * |  | * | * | * | * | *8* |
| Peng 2023 | * | * |  | * | ** | * | * | * | *8* |
| Ma 2014 | * | * | * | * | ** | * | * | * | *9* |
| Ma 2010 | * |  |  | * | ** | * | * | * | *7* |
| Lung 2013 | * | * | * |  | ** | * | * | * | *8* |
| Lu 2018 | * | * | * |  | ** | * | * | * | *8* |
| Liu 2016 | * | * | * | * | ** | * | * |  | *8* |
| Li 2018 | * | * | * | * | * | * | * | * | *8* |
| Lee 2017 | * | * | * | * | ** | * | * |  | *8* |
| Kuo CY 2022 | * | * | * | * | * | * | * | * | *9* |
| Kong 2016 | * |  | * | * | ** | * | * | * | *8* |
| Kim YJ 2017(1) | * | * | * |  | * | * | * | * | *7* |
| Kim YJ 2017(2) | * |  | * | * | ** | * | * |  | *7* |
| Kim JH 2017 | * | * | * |  | ** | * | * | * | *8* |
| Kawai 2013(1) | * | * | * |  | ** | * | * | * | *8* |
| Kawai 2013(2) | * | * | * | * | * | * | * | * | *8* |
| Kawai 2012 | * | * | * | * | * | * | * |  | *7* |
| Jiang 2023 |  | * | * | * | ** | * | * | * | *8* |
| Ishiguro 2017 | * | * | * | * | ** | * | * | * | *9* |
| Hu 2023 | * | * | * | * | * | * | * | * | *9* |
| He 2022 | * | * | * | * | * | * |  | * | *8* |
| Han 2016(1) | * | * | * | * | ** | * | * | * | *9* |
| Han 2016(2) | * | * | * | * | ** | * | * | * | *9* |
| Guo 2022 | * | * | * |  | * | * | * |  | *7* |
| Feng 2016 | * | * | * | * | ** | * | * | * | *9* |
| Eun 2023 | * | * | * | * | ** | * | * |  | *8* |
| Cheong 2016 | * | * | * | * | ** | * | * | * | *9* |
| Chen LL 2018 | * | * | * |  | ** |  | * | * | *7* |
| Chen 2023 | * | * | * | * | ** |  | * | * | *9* |
| Chen 2020 | * | * | * | * | * | * | * |  | *7* |
| Chen 2018 |  | * | * |  | * | * | * | * | *6* |
| Chen 2017 |  | * | * |  | * | * |  | * | *7* |
| Cardinale 2013 | * | * | * | * | ** | * |  | * | *7* |
| Li 2017 |  | * | * |  | ** | * | * | * | *7* |

A maximum of one star (*)*: can be given for each numbereditem within the ‘Selection' and ‘Outcome' a categories. While a maximum of two stars**: can be given for 'Comparability'.
